# Inactivation of p53 Is Sufficient to Induce Development of Pulmonary Hypertension in Rats

**DOI:** 10.1371/journal.pone.0131940

**Published:** 2015-06-29

**Authors:** S. Jacquin, V. Rincheval, B. Mignotte, S. Richard, M. Humbert, O. Mercier, A. Londoño-Vallejo, E. Fadel, S. Eddahibi

**Affiliations:** 1 INSERM U999, Centre Chirurgical Marie Lannelongue, Le Plessis Robinson, France; 2 Laboratoire de Génétique et Biologie Cellulaire, Université de Versailles Saint-Quentin-en-Yvelines, Montigny-le-Bretonneux, France; 3 INSERM U1046, Centre Hospitalier Universitaire Arnaud de Villeneuve, Montpellier, France; 4 Telomeres & Cancer laboratory, labeled “Ligue contre le Cancer”, UMR3244, Institut Curie, Paris, France; 5 Centre Chirurgical Marie Lannelongue, Le Plessis-Robinson, France; University of Giessen Lung Center, GERMANY

## Abstract

**Objective:**

Pulmonary artery smooth muscle cells (PA-SMCs) in pulmonary arterial hypertension (PAH) show similarities to cancer cells. Due to the growth-suppressive and pro-apoptotic effects of p53 and its inactivation in cancer, we hypothesized that the p53 pathway could be altered in PAH. We therefore explored the involvement of p53 in the monocrotaline (MCT) rat model of pulmonary hypertension (PH) and the pathophysiological consequences of p53 inactivation in response to animal treatment with pifithrin-α (PFT, an inhibitor of p53 activity).

**Methods and Results:**

PH development was assessed by pulmonary arterial pressure, right ventricular hypertrophy and arterial wall thickness. The effect of MCT and PFT on lung p53 pathway expression was evaluated by western blot. Fourteen days of daily PFT treatment (2.2 mg/kg/day), similar to a single injection of MCT (60 mg/kg), induced PH and aggravated MCT-induced PH. In the first week after MCT administration and prior to PH development, p53, p21 and MDM2 protein levels were significantly reduced; whereas PFT administration effectively altered the protein level of p53 targets. Anti-apoptotic and pro-proliferative effects of PFT were revealed by TUNEL and MTT assays on cultured human PA-SMCs treated with 50 μM PFT.

**Conclusions:**

Pharmacological inactivation of p53 is sufficient to induce PH with a chronic treatment by PFT, an effect related to its anti-apoptotic and pro-proliferative properties. The p53 pathway was down-regulated during the first week in the rat MCT model. These *in vivo* experiments implicate the p53 pathway at the initiation stages of PH pathogenesis.

## Introduction

Pulmonary arterial hypertension (PAH) is a rare and severe disease in which pressure elevation in the pulmonary arteries (PA) leads to right heart failure and death. There is no cure for PAH, and the effects of available therapeutic options are restricted to partial improvement of symptoms with a limited increase in survival. PAH can develop as an idiopathic disease (iPAH), or more frequently in association with underlying diseases, with an estimated incidence of 1 to 2 cases per million per year [1]. Arterial wall remodeling is the hallmark of severe advanced PAH. The central cellular processes underlying this vascular remodeling include an increase of pulmonary artery smooth muscle cell (PA-SMCs) growth and resistance to apoptosis [2,3]. Although the fundamental cause remains elusive, many disease-predisposing and function-modifying features of PA-SMCs have been identified. These include inflammation, cross-talk with pulmonary artery endothelial cells (PA-ECs), and *BMPRII* gene mutations [4,5]. On the other hand, several studies indicate that PA-SMCs from iPAH patients express abnormal phenotypes *ex vivo*, when separated from the influence of endothelial and inflammatory cells. These reports suggest intrinsic alterations in PA-SMCs characteristics resulting in dysfunctions in the signaling pathways that control cell cycle progression and proliferation.

Moreover, cellular phenotypic similarities between PAH and cancer have been proposed[6–9]. Indeed, pulmonary vascular remodeling observed in PAH is associated with hyper-proliferation, hypertrophy and distal extension of PA-SMCs, resistance to apoptosis, mitochondrial dysfunction with a “Warburg metabolic phenotype”, genomic instability and expression of cancer biomarkers [4,10,11].

Owing to the tumor suppressive properties of p53 [12,13], this protein has recently sparked interest in the PAH community. Mizuno et al. demonstrated that p53 knockout mice developed a more severe PH in response to chronic hypoxia compared to wild-type mice [14]. More recently, Mouraret et al. observed that Nutlin-3a, a MDM2 inhibitor [15], protected mice in different models of experimental pulmonary hypertension (PH) [16]. However, the consequence of pharmacological direct inactivation of p53 on the PH development has not been investigated. To clarify this, we chose to use the monocrotaline (MCT)-induced rat model of PH, which causes marked alterations in PA structure, including medial hypertrophy and intimal fibromuscular hyperplasia, similar to those observed in some forms of human PH.

In this work, we examined if inhibition of p53 activity by pifithrin-α (PFT) could induce pulmonary vascular remodeling and/or aggravate the MCT-induced PH model in rats. We evaluated lung p53 pathway protein expression during the development of MCT-induced PH and in response to PFT. The effect of PFT on the growth/apoptosis balance in isolated human PA-SMCs was also investigated.

## Methods

### Ethics statement

#### Animal

Experiments were performed in adult male Wistar rats (200 to 250 g) (Charles River, L’Arbresle, France) according to institutional guidelines that comply with European Union regulations (Directive 2010/63/EU of the European Parliament and the Council of 22 September 2010 for the protection of animals used for scientific purposes). The animal facility is licensed by the French Ministry of Agriculture (agreement N° B92-019-01). This study was approved by the local institutional animal experiments committee CEEA26 CAPSud and all experiments were supervised by Dr. Olaf Mercier (agreement by the French Ministry of Agriculture for animal experiments N° A92-396). Animals were housed in a temperature regulated room (12h/12h day/night cycle) with access to food and water *ad libitum* and placed 3 per cage. Rats were anesthetized with ketamine (60 mg/kg i.p., MTC Pharmaceuticals, Cambridge, Canada) and xylazine (4 mg/kg i.p., Bayer, Germany) and all efforts were made to minimize suffering. The sacrifice of animals resulted from the removal of heart and lungs after the measurement of pulmonary arterial pressure. All animal experiments reported are in accordance with the ARRIVE guidelines.

#### Human

The assays were performed on human PA-SMCs isolated from lungs obtained during lobectomy or pneumonectomy for localized lung cancer, collected by the anatomo-pathologist of the Marie Lannelongue chirurgical center (Le Plessis Robinson, France) at a distance from the tumor loci and considered as control cells without tumoral characteristics. This study was approved by the local ethics committee (CPP Ile-de-France VII, Le Kremlin-Bicêtre, France), has been conducted according to the principles expressed in the Declaration of Helsinki and all patients provided written informed consent before the study.

### Animal models and experimental design

In the first part of the study, pulmonary expression of p53 was examined in rats at various times after a single subcutaneous (s.c.) injection of MCT (60 mg/kg in HCl 1N, NaOH 1N and PBS, Sigma-Aldrich, Saint-Quentin-Fallavier, France): at day 1, day 3, day 7, day 14 and day 21. In the second part, to assess the pathophysiological consequences of pharmacological p53 activity inhibition, we assigned rats at random to 1 of 4 groups (5 animals in each group): two groups received daily intraperitoneal (i.p.) injection of PFT (2.2 mg/kg/day in DMSO 1% NaCl, Interchim, Montluçon, France); two groups received vehicle. Treatments were given for two weeks after a single MCT injection or after an injection of vehicle. PH development and pulmonary expression of p53 pathway proteins were then evaluated in all rats.

### Assessment of PH

After rats anesthesia, a polyvinyl catheter was introduced into the right jugular vein and pushed through the right ventricle into the PA. After measurement of pulmonary arterial pressure (PAP) with LabChart software (ADInstruments, USA), the thorax was opened and the left lung immediately removed and frozen for p53 expression analysis. The heart was dissected and weighed for calculation of the right ventricular hypertrophy index (ratio of right ventricular free wall weight divided by the sum of the septum plus left ventricular free wall weight (RV/LV+S)). The right lung was fixed in the distended state with intratracheal infusion of formalin buffer. After paraffin embedding, 5-μm-thick lung sections were mounted on Superfrost slides and stained with hematoxylin-eosin. For each rat, 40 to 60 intra-acinar arteries were analyzed and categorized as fully muscularized (M), partially muscularized (PM) or non-muscularized (NM) to assess the degree of muscularization.

### Evaluation of in situ PA-SMCs proliferation, matrix accumulation and macrophages infiltration

To assess PA-SMCs proliferation in rat pulmonary arteries, proliferating cell nuclear antigen (PCNA) staining was performed. Tissue sections were deparaffinized in toluene and then treated with a graded series of ethanol washes, rehydrated in TBS (pH 7.5), and incubated with target retrieval solution (citrate pH6) in a pressure cooker. Slides were then washed in TBS, incubated for 30 minutes in a protein-blocking solution (goat serum 10% in PBS), and incubated for 1 hour with an anti-PCNA mouse monoclonal antibody (M0879, clone PC-10, 1:200, Dako, Les Ulis, France) in the presence of streptavidin/biotin endogenous blocking reagents (SP-2002, Vector, Burlingame, USA). The slides were then incubated with a mouse biotinylated secondary antibody for 30 minutes, followed by an amplification step with the Vectastain ABC-AP Kit (AK-5002, Vector) for 30 minutes. The revelation was processed with the Vector Red Alkaline Phosphatase Substrate Kit (SK-5100, Vector), and nuclei were counterstained with hematoxylin.

To analyze matrix accumulation, a Masson trichrome stain using hematoxylin (nuclear staining), ponceau fuchsin (cytoplasm staining) and anyline blue (collagen staining) was performed on lungs sections.

The infiltration of macrophages was evaluated by immunostaining using a mouse monoclonal anti-CD68 antibody (MCA341R, AbDserotec, Colmar, France) revealed by a 594-Alexa fluor anti-mouse secondary antibody. Smooth muscle fibers were stained with a mouse monoclonal anti-α-Smooth Muscle Actin (SMA) FITC conjugated antibody (F3777, Sigma-Aldrich). Nuclei were stained with DAPI.

At the end of the procedure, all stainings were analyzed with an epifluorescence microscope and NiS element software (Nikon 80i, Japan).

### Western blot

Western blot assays were performed on rat lungs stored at -80°C. Samples were lysed in buffer containing phosphatases and proteases inhibitors. Total proteins (100 μg) were separated on 4–12% NuPage Bis-Tris gels (Life Technologies, Carlsbad, USA) and transferred onto PVDF membranes (GE Healthcare, Freiburg, Germany). The membranes were incubated over-night at 4°C with a primary antibody: mouse monoclonal anti-p53 (clone PAb122, LS-C63152, LSBio, Seattle, USA), rabbit polyclonal anti-p21 (sc-397, Santa Cruz, Le Perray en Yvelines, France), rabbit polyclonal anti-Bax (sc-493, Santa Cruz), mouse monoclonal anti-MDM2 (MCA1709, AbDserotec) or mouse monoclonal anti-βactin (clone AC-74, A5316, Sigma-Aldrich). Incubation with a secondary HRP antibodies (anti-mouse sc-2314 or anti-rabbit sc-2313, Santa Cruz) was performed for 1 hour at room temperature. HRP signals were detected by ECL substrate (Immun-Star WesternC Kit, Biorad, Marnes-la-Coquette, France) using a ChemiDoc XRS+ (Biorad) and blots were quantified and normalized by actin signal with ImageLab software (Biorad).

### Human PA-SMCs isolation and culture

Human PA-SMCs were isolated and cultured as previously described [17]. Assays were performed on cells at passages 3–6. PA-SMCs were treated for 24 hours with increasing doses of PFT (0,1 μM to 50 μM, Interchim), in the presence or absence of 200 μM etoposide (Sigma-Aldrich) or 150 μM hydrogen peroxide (H_2_O_2_, Sigma-Aldrich).

### PA-SMCs proliferation-viability assay

The effect of PFT on the PA-SMCs proliferation-viability of PA-SMCs was assessed by MTT assays. Briefly, cells were seeded in 96-wells culture plates with 10^5^ cells per well for 24 hours in medium supplemented with 10% FBS, followed by a FBS deprivation for 48 hours to induce growth arrest. Cells were then treated for 24 hours in medium supplemented with 10% FBS. MTT tetrazolium dye was added and after 4 hours incubation at 37°C, the colored formazan formation dissolved in DMSO was analyzed by a spectrophotometer at 570 nm (microplate reader Model 680, Biorad). All samples were analyzed in triplicate.

### PA-SMCs apoptosis detection

Apoptosis of PA-SMCs was evaluated by immunostaining with TUNEL (TdT-mediated dUTP Nick-End Labeling) assays. Briefly, paraformaldehyde-fixed cells grown on coverslips were incubated with TUNEL reaction mixture in a humidified atmosphere for 1 hour at 37°C in the dark according to the manufacturer’s instructions (DeadEnd TUNEL System, Promega, Madison, USA). Cells on coverslips were then counterstained with DAPI and examined with an epifluorescence microscope and NiS element software (Nikon 80i, Japan). Quantification of TUNEL positives cells were performed with 4 images per coverslips.

### Statistics

Statistics were performed on GraphPad Prism 5 software and data are expressed as means ± SEM. Gaussian distributions of values were tested with adequate normality tests. Data that followed Gaussian distribution were analyzed with parametric tests, such as ANOVA associated with Dunnett post-tests to compare three or more groups to the control group and t-tests to compare two groups. Tukey post-tests were performed for statistical comparisons of the three treated groups in the study evaluating the effect of PFT on rat PH development. Matched ANOVA or paired t-tests were used when values are paired. Data that did not follow Gaussian distribution were analyzed with non parametric tests (Mann-Whitney tests).

## Results

### Development of MCT-PH is associated with an early p53 decrease

**Fig 1:**
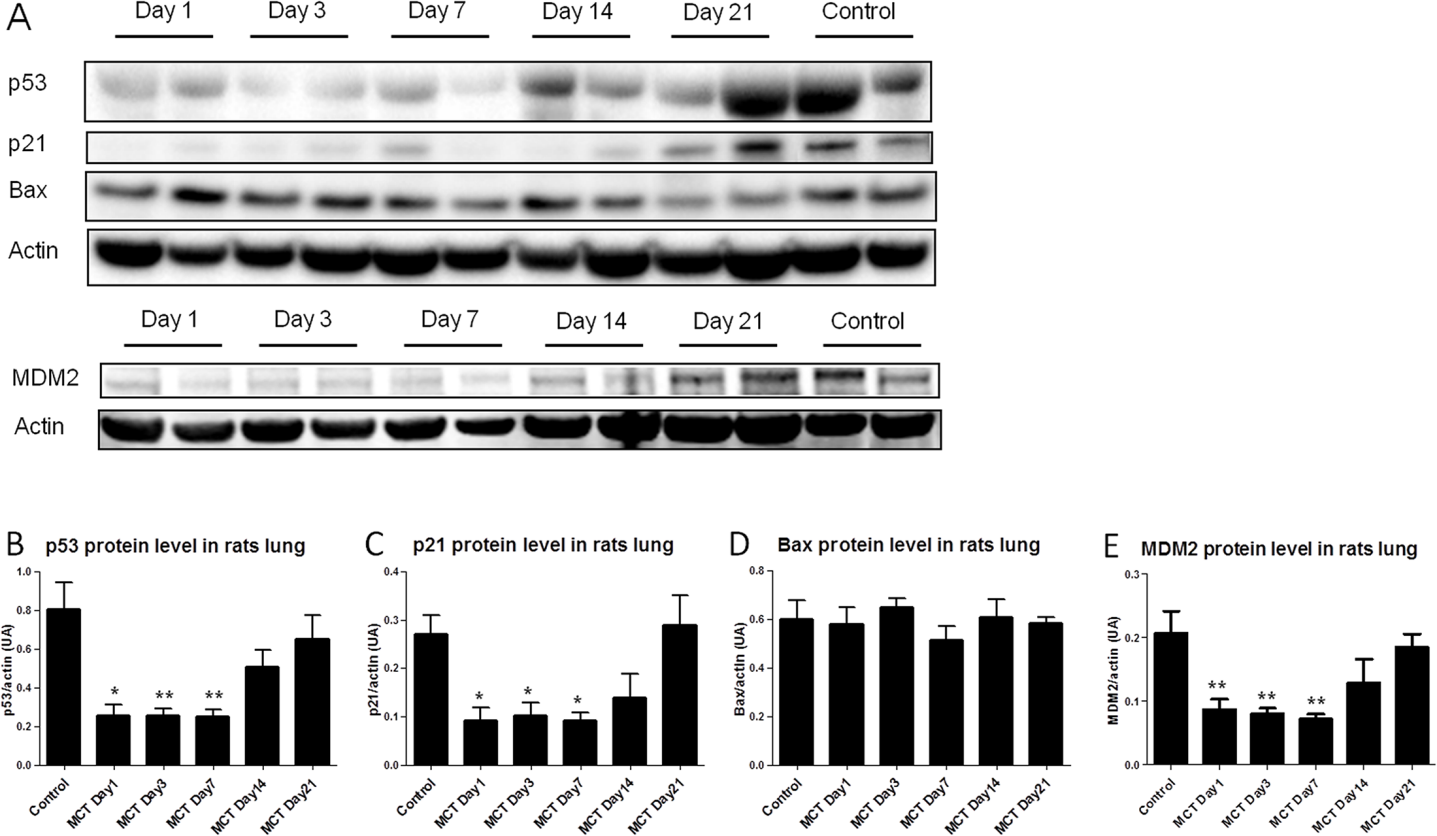
Expression of p53 and its targets in rat lungs during the development of MCT-PH. Lungs were removed at day 1, day 3, day 7, day 14 and day 21 after the monocrotaline injection (day 0) (5 animals per group). A: Representative western immunoblots of p53 (55 kDa), p21 (21 kDa), Bax (21 kDa), MDM2 (90 kDa) and βactin (42 kDa) protein levels in rat lungs. B-E: Quantification of p53, p21, Bax and MDM2 protein levels from western blots analysis. Values represent mean ± SEM of the protein level / actin level quantification (n = 5). * p<0.05; ** p<0.01 vs. Control group (Mann-Whitney tests).

In order to determine the temporal expression of p53 in PH development, the lung protein level was measured at various times after MCT injection. The measurements were performed in five animals for each group. The results showed an early and dramatically decrease of p53 protein level within the first 24 hours, which was maintained during the first week compared to controls receiving vehicle (day 1, p = 0.0159; day 3, p = 0.0079 and day 7, p = 0.0079) (Fig 1B). Then p53 protein expression was gradually increased and normalized by the second week. This result revealed that deregulation of p53 is early and arises in the initiation stage of PH development in the MCT rat model. Similarly, the protein level of p21, a transcriptional target of p53 implicated in cell cycle arrest, was significantly decreased during the first week compared to controls (day 1, p = 0.0317; day 3, p = 0.0159 and day 7, p = 0.0159) (Fig 1C). In contrast, no difference in BAX protein level was found in comparison to control animals (Fig 1D). We also measured the protein level of MDM2, the major regulator of p53 and also a transcriptional target of p53. Similarly to p53, the MDM2 protein level was decreased during the first week compared to the value obtained in controls (day 1, p = 0.0095; day 3, p = 0.0043 and day 7, p = 0.0095) (Fig 1E).

### PH induction by pharmacological p53 inactivation with PFT in rats

**Fig 2:**
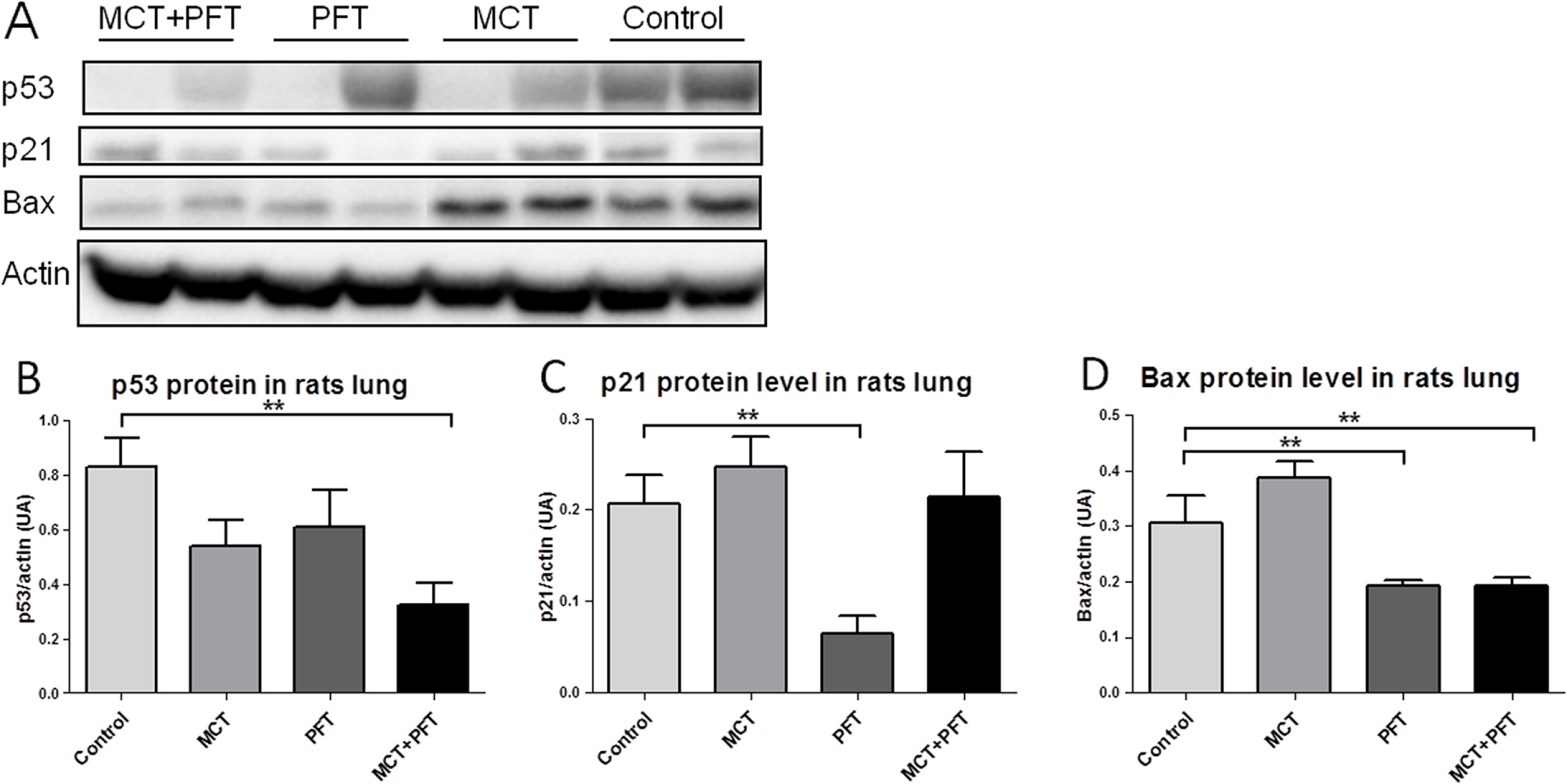
Effective inhibition of p53 activity with PFT in rat lungs. Effect of chronic PFT treatment on p53, p21 and Bax protein levels in rat lungs (5 animals per group). A: Representative western immunoblots of p53 (53 kDa), p21 (21 kDa), Bax (23 kDa) and βactin (42 kDa) protein levels in rat lungs. B-D: Quantification of p53, p21 and Bax western blots. Values represent mean ± SEM of the protein level / actin level quantification (n = 5). ** p<0.01 vs. Control group (Mann-Whitney tests).

To validate the effect of PFT on p53 activity, western blots were performed on lungs samples collected at the end of 14 days of treatment (5 animals per group). As shown in Fig 2C and 2D, PFT rats treatment decreased both p21 and BAX protein level compared to the control group (p = 0.0080 and p = 0.0079, respectively). These results support the effective inhibition of p53 activity with PFT treatment. Moreover, as illustrated in Fig 2B, the p53 protein level was decreased significantly in the MCT+PFT group compared to the control group (p = 0.0025), suggesting a possible synergic effect of the two compounds.

**Fig 3:**
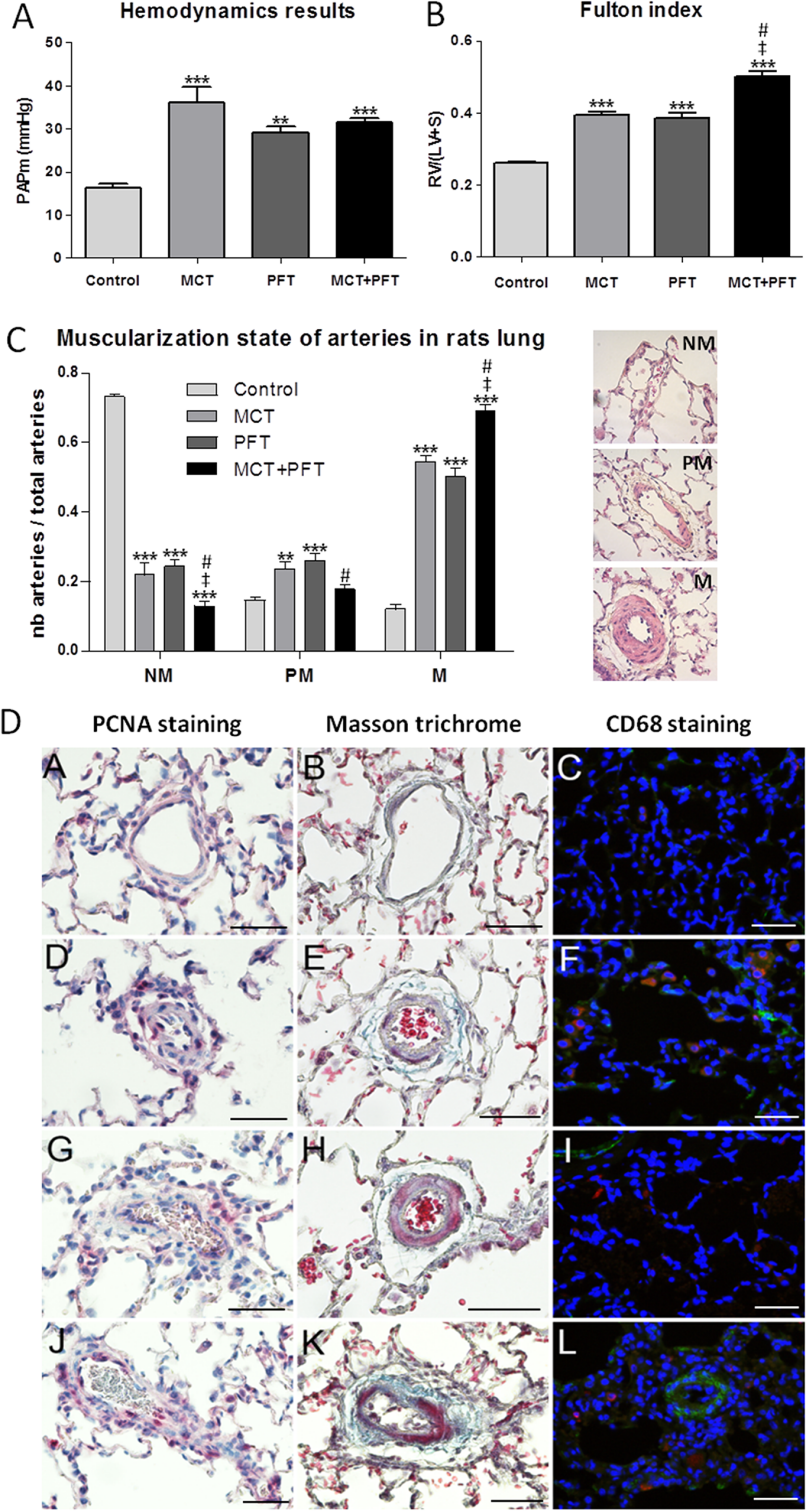
Consequence of p53 inactivation with PFT on PH development in rats. A: Hemodynamic measurements (PAPm values in mmHg) obtained in rats treated respectively with MCT, PFT, MCT+PFT or vehicle (corresponding to Control group). B: Right ventricular hypertrophy estimated by the right ventricle / left ventricle+septum weight ratio (Fulton index). C: Illustration and quantification of the muscularization state of pulmonary arteries categorized as non-muscular (NM), partially muscular (PM) and muscular (M) according to the media thickness as determined by hematoxylin-eosin staining. Values are mean ± SEM (n = 5). Statistics: ** p<0.01; *** p<0.001 vs. Control group (Dunnett post-tests). ‡ p<0.05 vs. MCT group and # p<0.05 vs. PFT group (Tukey post-tests). D: Immunostaining on lung sections of rats treated with MCT (images D, E, F), PFT (images G, H, I), MCT+PFT (images J, K, L) or vehicle (images A, B, C) to detect: cell proliferation (PCNA staining, images A, D, G, J), matrix accumulation (Masson trichrome, images B, E, H, K) and macrophage infiltration (red CD68 staining combined to green αactin staining) (images C, F, I, L). Scale bars: 50 μm.

To determine whether inhibition of p53 activity would influence PH development alone and/or affect the MCT-induced PH, PFT was injected daily for 14 days in rats, with or without MCT administration at day 0. As compared to the control group, the MCT group showed a significant increase of PAP values (36 mmHg *vs*. 16.3 mmHg). When combined with PFT treatment, no difference was seen between the MCT and the MCT+PFT groups (36 mmHg *vs*. 31.6 mmHg) (Fig 3A). However, chronic rats treatment with PFT alone significantly increased PAP values compared to the control rats receiving vehicle (29 mmHg *vs*. 16.3 mmHg).

In agreement with hemodynamic parameters, the right heart hypertrophy index, also called Fulton index, and the muscularization of distal pulmonary arteries showed a significant increase in the MCT and the MCT+PFT groups but also in the PFT group (Fig 3B and 3C). Moreover, statistical comparisons of the three treated groups (Tukey post-tests) revealed that the combination of treatments (MCT+PFT) increased cardiac hypertrophy and PA muscularization compared to MCT and PFT given alone.

In line with the muscularization of distal pulmonary arteries, the PA-SMCs proliferation within the arterial wall (PCNA staining) and accumulation of collagen fibers (blue staining in Masson trichrome coloration) were increased in the PFT, MCT and MCT+PFT groups, compared to the control group (Fig 3D). However, infiltration of macrophages (red CD68 staining) was observed in the MCT group (Fig 3D–3F) and the MCT+PFT group (Fig 3D–3L) but not in the PFT group (Fig 3D–3I), suggesting that PFT did not interfere with the inflammatory process.

### Effects of PFT on human PA-SMCs growth and apoptosis

**Fig 4:**
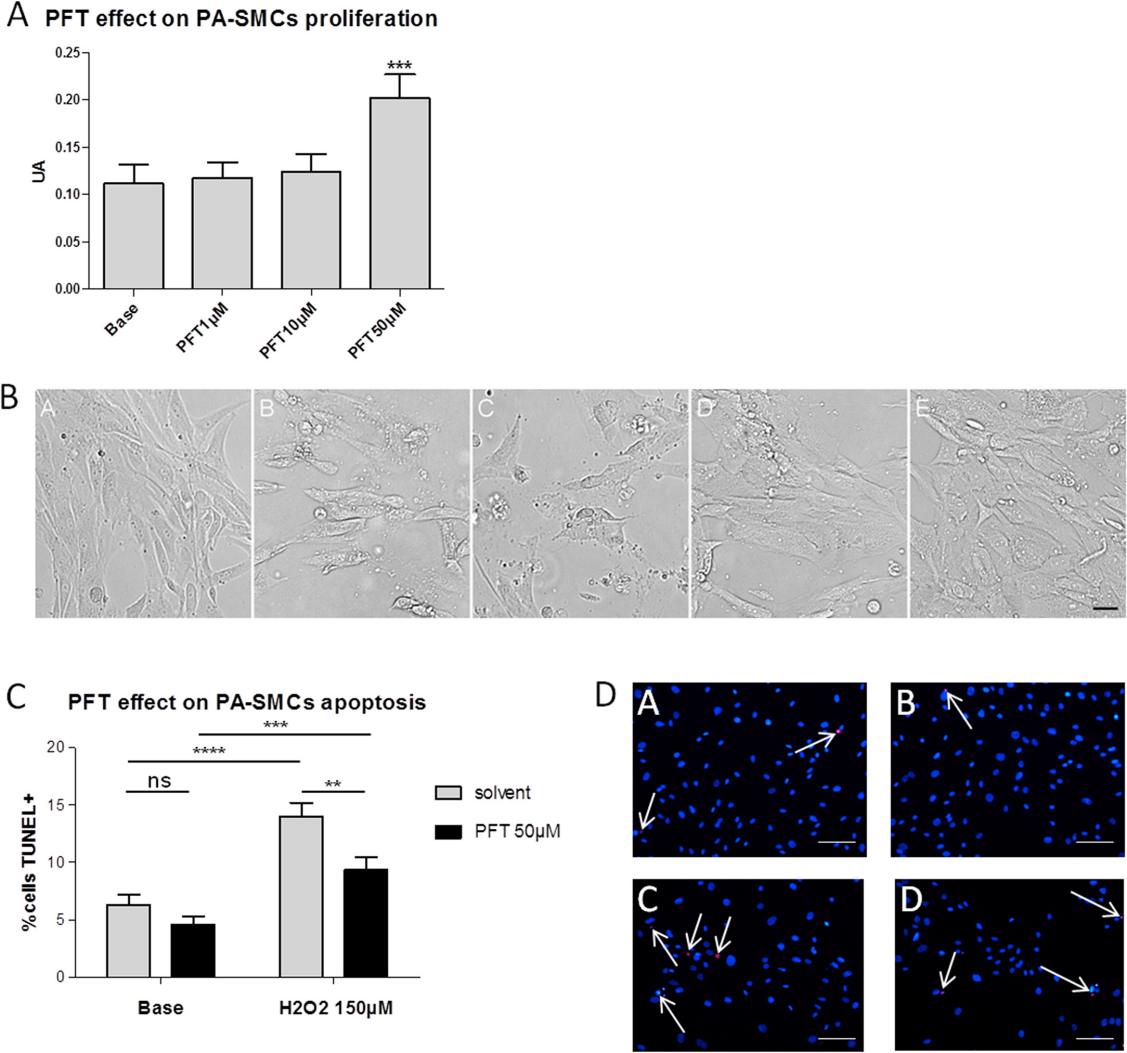
Pro-proliferative and anti-apoptotic effects of PFT in human control PA-SMCs. A: Growth of PA-SMCs in response to increasing doses of PFT (1, 10, 50 μM), evaluated by MTT assays. Values are means ± SEM of 4 experiments. *** p<0.001 vs. corresponding Base (Dunnett post-tests). B: Illustrations of the morphological protective effect of increasing doses of PFT (1 μM: image C, 10 μM: image D, 50 μM: image E) against cellular death induced by etoposide (image B). Image A = non treated condition. Scale bar: 37,5 μm. C: Effect of PFT treatment (50 μM) on PA-SMCs apoptosis induced by H2O2 (150 μM) evaluated by TUNEL assays. The ratio of TUNEL positives cells / total cells was calculated on each picture (24 pictures (from 6 different patients) in basal condition and 16 pictures (from 4 different patients) in H_2_O_2_ condition) and values are means ± SEM (expressed in percentages).** p<0.01; *** p<0.001; ****p<0.0001 (unpaired t-tests). D: Representative images of apoptosis assays with red TUNEL staining and blue DAPI staining. Images A and C illustrate the solvent conditions, respectively without and with 150 μM H202 treatment. Images B and D illustrate the 50 μM PFT conditions, respectively without and with 150 μM H202 treatment. Scale bars: 50 μm.

To evaluate the direct consequences of p53 inactivation by PFT on pulmonary vascular cells growth and apoptosis, we first tested the dose-dependent effect of PFT on human PA-SMCs growth with MTT assays. As illustrated in Fig 4A and 4B, PFT increased PA-SMCs growth at 50 μM and protected cells against cell death induced by etoposide, a cellular stress known to induce p53 (Fig 4B–4E). Owing to these first results, we used the 50 μM concentration to evaluate the effect of PFT on PA-SMCs apoptosis with TUNEL assays. As shown in Fig 4C and 4D, PFT had an anti-apoptotic effect on PA-SMCs, an effect which was more marked in the presence of H_2_O_2_, a well-known apoptosis inducer. We tested increasing doses of H_2_O_2_ and 150 μM was associated with a maximum of apoptosis in this assay. At this dose, a significant increase of TUNEL positives cells was shown in presence of H_2_O_2_ (p<0.0001, Fig 4DA *vs*. Fig 4DC) and the number of TUNEL positives cells decreased significantly with PFT in this apoptotic condition (p = 0.0078, Fig 4DC *vs*. Fig 4DD). In basal condition, PFT trended to decrease the number of TUNEL positives cells (p = 0.1497, Fig 4DA *vs*. Fig 4DB).

## Discussion

Our study demonstrates that MCT-induced PH in rats is associated with an early decrease of p53 expression, which occurs prior to PH development. This reveals that down-regulation of p53 could be an initiating phenomenon of PH pathogenesis. Indeed pathological hemodynamics changes occur from the 14th day after MCT administration [18]. Similarly, the down-expression of the transcriptional p53 target p21 was also observed in the first week, indicating a deregulation of cell cycle at this stage, allowing the initiation of an excessive pulmonary cellular proliferation, leading to the development of PH. However, MCT seems to have no effect on BAX protein levels in rat lungs in the studied stages suggesting that p53-BAX pathway was not involved in the pathogenesis of PH in this experimental model. Also, we did observe a significant decrease of MDM2 protein level, the main p53 regulator [19,20]. These results suggest that MCT-induced PH is associated to a direct alteration of p53 expression and regulation.

Fourteen days after MCT administration, when PH was established, the expressions of p53, p21 and MDM2 were normalized. These results suggest that the alteration of the p53 pathway play a key role at the initiation stage of MCT-induced PH but not at later stages. Our data are consistent with those of Mouraret et al. in which no difference in p53 protein level was seen between chronic hypoxia-induced PH mice and control mice [16].

Because the mechanism by which MCT-induced PH is complex[21], and to investigate the direct role of p53 in the pathogenesis of PH, we chose to investigate the consequence of the inhibition of p53 activity by using PFT, which was originally developed to reduce side-effects of aggressive cancer therapies [22]. Interestingly, in our rat model, daily administration of PFT for two weeks not only aggravated the MCT-induced PH, but the drug alone was sufficient to induced PH, revealed by an increase of PAP, right cardiac hypertrophy and small pulmonary arteries muscularization. This effect was related to in situ PA-SMCs proliferation and accumulation of collagen. These results suggest that the inactivation of p53 alone might be a trigger of PH. This conclusion is not consistent with the work of Mizuno et al. in which the knockdown of p53 in mice was not associated with the development of a spontaneous PH under normoxic environment, though the knockout mice did develop a more severe PH than wild-type mice in response to chronic hypoxia [14]. These differences may be related to the species and or to the compensatory mechanisms often observed in transgenic models.

In both human and experimental PH, PA-SMCs play a central role in the development and progression of the disease. Recently we demonstrated the relationship between telomere length, p53 and excessive PA-SMCs growth in iPAH. Here we investigated the pharmacological p53 inactivation by PFT on PA-SMCs growth and resistance to apoptosis. Clearly, cells treatment with 50 μM PFT increased PA-SMCs growth under basal conditions and protected cells from death induced by etoposide, known to induce p53 activation. Moreover, PFT protected PA-SMCs against apoptosis induced by H_2_O_2_. Thus, PFT cells treatment induced a phenotype similar to those presented by PA-SMCs from iPAH patients, revealing the important effect of p53 inactivation in PH characteristics.

In conclusion, this study has clearly demonstrated the key role of p53 in maintaining the balance of human PA-SMCs growth/apoptosis and in the initiation stage of PH development in the MCT model. Moreover, we have identified that the inactivation of p53 by pharmacological means is sufficient to induce the development of experimental PH.
